# Acute Pain Burden and Opioid Dose Requirements after Cesarean Delivery in Parturients with Preexisting Chronic Back Pain and Migraine

**DOI:** 10.1155/2021/3305579

**Published:** 2021-08-30

**Authors:** Ryu Komatsu, Michael G. Nash, Kenneth C. Ruth, William Harbour, Taylor M. Ziga, Shane Mandalia, Emily M. Dinges, Davin Singh, Hani El-Omrani, Joseph Reno, Brendan Carvalho, Laurent A. Bollag

**Affiliations:** ^1^Department of Anesthesiology and Pain Medicine, University of Washington, 1959 NE Pacific Street, Seattle, WA 98195, USA; ^2^Department of Statistics, University of Washington, 1959 NE Pacific Street, Seattle, WA 98195, USA; ^3^Department of Anesthesiology, Perioperative and Pain Medicine, Stanford University School of Medicine, 453 Quarry Road, Stanford, CA 94304, USA

## Abstract

**Introduction:**

Preexisting chronic pain has been reported to be a consistent risk factor for severe acute postoperative pain. However, each specific chronic pain condition has unique pathophysiology, and it is possible that the effect of each condition on postoperative pain is different.

**Methods:**

This is a retrospective cohort study of pregnant women with preexisting chronic pain conditions (i.e., migraine, chronic back pain, and the combination of migraine + chronic back pain), who underwent cesarean delivery. The effects of the three chronic pain conditions on time-weighted average (TWA) pain score (primary outcome) and opioid dose requirements in morphine milligram equivalents (MME) during postoperative 48 hours were compared.

**Results:**

The TWA pain score was similar in preexisting migraine and chronic back pain. Chronic back pain was associated with significantly greater opioid dose requirements than migraine (12.92 MME, 95% CI: 0.41 to 25.43, *P*=0.041). Preoperative opioid use (*P* < 0.001) was associated with a greater TWA pain score. Preoperative opioid use (*P* < 0.001), smoking (*P*=0.004), and lower postoperative ibuprofen dose (*P*=0.002) were associated with greater opioid dose requirements.

**Conclusions:**

Findings suggest women with chronic back pain and migraine do not report different postpartum pain intensities; however, women with preexisting chronic back pain required 13 MME greater opioid dose than those with migraine during 48 hours after cesarean delivery.

## 1. Introduction

Cesarean delivery is the most common inpatient surgery in the United States with 1.3 million performed annually. Severe acute postpartum pain is a strong risk factor for chronic postcesarean pain, [1, 2], and estimated 1 in 300 opioid-naïve women becomes persistent opioid users after cesarean delivery [3]. Pain control is the leading maternal preference prior to cesarean delivery [4], and poorly controlled postpartum pain predisposes to postpartum depression [5].

There have been considerable efforts made to elucidate risk factors for severe acute pain and increased analgesic requirement after multiple types of surgeries. Along with female sex [6, 7] and younger age [6–9], preexisting chronic pain has been reported to be the most consistent risk factor for severe acute postoperative pain [6, 9–12]. Preexisting chronic pain has been defined in previous studies as the existence of pain as a dichotomous parameter (yes/no) [9, 10, 12] or by pain intensity measured by the numeric rating scale [6, 11], and information regarding the influence of specific chronic pain conditions on severity of acute postoperative pain is lacking. However, each chronic pain condition has unique pathophysiology and symptomatology, and it is possible that the effect of each condition on postoperative pain is different.

We performed a retrospective cohort study to compare opioid consumption and pain intensity during the acute postoperative period after cesarean delivery between patients who had different types of chronic pain conditions that are most common in women of child-bearing age (i.e., migraine and chronic back pain). Our hypotheses were that (1) pain intensity (primary outcome) and (2) opioid dose requirements during 48 postoperative hours would differ between patients with different chronic pain conditions.

## 2. Materials and Methods

After institutional review board approval and a waiver of consent (University of Washington IRB ID: STUDY00011433, approval date: 10/15/2020), we obtained data from women aged 18 or older with documented preexisting chronic pain conditions (i.e., migraine, chronic back pain, or combination of migraine and chronic back pain), who underwent cesarean delivery at the University of Washington Medical Center between May 2012 and October 2020. Only cesarean deliveries under neuraxial anesthesia (i.e., spinal, epidural, or combined spinal-epidural anesthesia) receiving intrathecal or epidural morphine administration were included. Exclusion criteria included the following: women aged less than 18 years, cesarean deliveries performed under general anesthesia, those who received postoperative epidural infusions, and women who received mechanical ventilation with sedation postoperatively. Women who had chronic pain conditions other than migraine and chronic back pain (i.e., fibromyalgia, pelvic pain, multiple sclerosis, and sickle cell disease) were also excluded. During the study period of 2012 to 2020, postoperative order set consisting of scheduled acetaminophen 1000 mg every 6 hours and scheduled ibuprofen 600 mg every 6 hours, with oxycodone 5 to 10 mg every 3 hours as needed for moderate-to-severe pain, supplemented by intravenous morphine 2 mg one time as needed for breakthrough pain, was used at the University of Washington Medical Center. If pain was not controlled after the administration of intravenous morphine, the patients were evaluated by the anesthesia team for further management. This study followed the Strengthening the Reporting of Observational Studies in Epidemiology (STROBE) reporting guidelines.

Time-weighted average (TWA) pain score during 48 postoperative hours was the primary outcome. Postoperative pain was evaluated using a verbal numerical rating scale (0–10) (0 = no pain; 10 = worst pain imaginable) every 15 minutes up to 2 hours after surgery in the postanesthesia recovery unit and then every 4 hours for 48 hours by nurses. The pain score reported was at the body locations relevant for postcesarean delivery (i.e., incisional pain, abdominal pain, and pelvic pain). TWA pain score was equal to the sum of the portion of each time interval in between two adjacent pain score measurements multiplied by the average of the corresponding two pain scores that was then divided by the time interval between the first and the last pain score measurements [13, 14]. Therefore, it could be calculated even if the pain scores were not reported every 4 hours or the pain scores were not reported for certain periods of time [14]. TWA pain score was calculated from all the available pain scores during the postoperative 48-hour study period.

Opioid dose requirements during 48 postoperative hours were the secondary outcome of the study, converted into morphine milligram equivalents (MME) utilizing the conversion table of the Multicenter Perioperative Outcomes Group (https://phenotypes.mpog.org/Oral%20Morphine%20Equivalent). Intrathecal or epidural morphine administered intraoperatively was not included in the opioid dose requirements during 48 postoperative hours.

Demographic and obstetric variables deemed biologically plausible to be associated with postoperative pain, and opioid dose requirements were retrieved from the medical records (Table 1).

## 3. Statistical Analyses

The effect of each chronic pain condition on each outcome was assessed using analysis of covariance (ANCOVA) with the covariates listed in Table 1. An indicator variable was added to the model to denote missing body mass index (BMI) values for five participants replaced with the mean observed BMI for the purpose of modeling the two outcomes.

The effects of migraine, chronic back pain, and the combination of migraine + chronic back pain on the TWA pain score were compared using Tukey's HSD test with ordinary least squares (OLS) ANCOVA to maintain alpha of 0.05. The effects of migraine, chronic back pain, and the combination of migraine + chronic back pain on the opioid dose requirements were compared using *t*-tests based on the estimated covariance matrix from the ANCOVA model using rank-based estimation with the Bonferroni correction to maintain alpha of 0.05. The rank-based estimation was used to fit the opioid dose requirement model [15, 16] as poor model fit was observed with OLS ANCOVA due to high influence by a relatively small number of individuals requiring unusually high opioid doses.

Coefficient estimates and Bonferroni adjusted 95% confidence interval (CI) were reported from the OLS ANCOVA model for the TWA pain score and the rank-based estimation ANCOVA model for opioid dose requirements. The effects of covariates (other than each chronic pain condition) on each outcome were evaluated using the Bonferroni method to maintain alpha of 0.01. R statistical software and SAS software (version 9.4; SAS Institute, Cary, NC, USA) were used for all analyses.

## 4. Results

Of 781 patients whose data were obtained, 465 had migraine, 273 had chronic back pain, and 43 had both migraine and chronic back pain (Table 1). TWA pain scores (mean (standard deviation (SD)) during 48 postoperative hours for patients with migraine, chronic back pain, and the combination of migraine and chronic back pain were 3.4 (1.4), 3.7 (1.6), and 4.1 (1.7), respectively. Opioid dose requirements in MME (median (interquartile range (IQR))) during 48 postoperative hours were 60 [23 to 120], 90 [37 to 156], and 105 [37 to 171], respectively (Table 2). Among 781 patients, 6 were on gabapentinoids, and 31 were on skeletal muscle relaxants preoperatively for the management of their chronic pain conditions, of whom 5 continued gabapentinoids and 10 continued skeletal muscle relaxants postoperatively.

No chronic pain condition (migraine, chronic back pain, or the combination of both) was associated with significantly different TWA pain scores than any other. Chronic back pain alone was associated with significantly greater opioid dose requirements than migraine alone (12.92 MME, 95% CI: 0.41 to 25.43, *P*=0.041). The combination of both conditions was associated with greater opioid dose requirement than migraine alone (10.39 MME, 95% CI: −15.47 to 36.24, *P* > 1) and slightly less than chronic back pain alone (2.53 MME, 95% CI: −28.40 to 33.47, *P* > 1), but neither difference was statistically significant.

Preoperative opioid use was associated with greater TWA pain scores (1.14, 95% CI: 0.42 to 1.87, *P* < 0.001). Preoperative opioid use (156 MME, 95% CI: 121 to 191, *P* < 0.001) and smoking (26.3 MME, 95% CI: 4.67 to 47.99, *P*=0.004) were associated with greater opioid dose requirements. Greater postoperative ibuprofen dose (4.34 MME per 600 mg, 95% CI: 0.91 to 7.77, *P*=0.002) was associated with lower opioid dose requirements.

## 5. Discussion

We found no significant differences in postoperative pain intensity during 48 postoperative hours with any of the chronic pain conditions (preexisting migraine, chronic back pain, and the combination of both migraine and chronic back pain) that were compared. However, the patients with preexisting chronic back pain required 12.9 MME greater opioid dose than those with migraine during 48 postoperative hours. The difference is equivalent to 1.7 5 mg oxycodone tablets and therefore appears to be clinically significant.

Different chronic pain conditions have been shown to have different pain perception profiles assessed by quantitative sensory testing (QST). QST consists of multiple modalities of stimulation (i.e., pressure, punctate, electrical, hot, and cold) and has been documented in neuropathic pain conditions including polyneuropathy, postherpetic neuralgia, peripheral nerve injury, complex regional pain syndrome, trigeminal neuralgia, and central pain [17]. Although no studies have made a direct comparison of QST profiles between migraine and chronic back pain, a meta-analysis of QST profiles of migraine patients and healthy control subjects demonstrated migraine patients had lower pressure pain detection thresholds in the head and neck area (painful body site) than healthy control subjects [18]. However, pressure pain detection thresholds in the nonpainful body site were not different from controls [18]. In contrast, a meta-analysis comparing QST profiles of patients with chronic low back pain and healthy control subjects showed lower pressure pain detection in nonpainful body sites compared with controls [19]. Therefore, there might be significant differences in QST profiles between those with migraine and chronic back pain. Since pressure pain detection threshold measured at a nonoperative site (i.e., finger) has been shown to be inversely correlated with postoperative 48-hour morphine requirement among cesarean delivery patients [20], the difference in QST profile between migraine and chronic back pain might translate into the difference in postoperative opioid dose requirements observed in the current study. The underlying mechanism for this is yet to be elucidated. Migraine has been known to worsen during the early postpartum period, probably triggered by abrupt fall in the level of estrogens. Chronic back pain can worsen during the postpartum period due to immobility and body position. The effects of dynamic changes in the chronic pain conditions during the postpartum period could also have confounded the postoperative opioid dose requirements. Among 6 patients who were on gabapentinoids for the management of chronic pain conditions preoperatively, 1 discontinued the medications postoperatively. And 21 among 31 patients who were on skeletal muscle relaxants preoperatively discontinued the medications postoperatively. Postoperative discontinuation of these nonopioid analgesic adjuvants potentially increases postoperative opioid requirements, but the effect size of this factor on the postoperative opioid dose requirement outcome is likely trivial due to very small number of this subgroup relative to overall sample size.

Our study demonstrated a nonstatistically significant trend of increased postoperative opioid dose requirements in patients who had both migraine and chronic back pain compared with those with only migraine. Whether the coexistence of multiple chronic pain conditions has an additive effect on postoperative pain intensity and opioid requirements is an area of future research. The nonsignificant difference in opioid dose requirements between patients with both chronic back pain and migraine and those with migraine only (10.4 MME) was similar in magnitude to the significant difference between patients with chronic back pain only and those with migraine only (12.9 MME). The former failed to reach statistical significance because the relatively small number of patients with both conditions in our sample resulted in reduced statistical power for this test.

Preoperative opioid use was associated with increased postoperative pain intensity and largely increased postoperative opioid dose requirements. With holding other covariates constant, a patient on opioids preoperatively was expected to have 1.14-point higher TWA pain score (on 1 to 10 scale) and require 156 MME higher opioid dose during 48 postoperative hours than a patient who was not on opioids preoperatively. Preoperative opioid use can induce opioid tolerance and opioid-induced hyperalgesia. One study demonstrated that patients with chronic opioid use required more than 3 times as much morphine given via epidural infusion than opioid-naïve patients [21]. Another study reported patients on chronic opioids experienced greater postoperative pain despite requiring 3-fold larger opioid doses than opioid-naïve patients [22].

Smoking history was associated with increased opioid dose requirements with a clinically significant effect size. With holding other covariates constant, a patient who has a history of smoking was expected to require 26.3 MME higher opioid dose during 48 postoperative hours than a patient who does not have a history of smoking. Several previous studies demonstrated higher acute postoperative pain scores and increased analgesic requirement in smokers after multiple types of surgery [23, 24]. This may be partially explained by nicotine withdrawal and altered nicotinic receptor availability. In acutely abstinent smokers, typical of the perioperative time frame, beta-2 nicotinic acetylcholine receptor availability is increased, which is associated with increased pain sensitivity [25].

Lower postoperative ibuprofen dose was associated with greater postoperative opioid dose requirements. Every 600 mg increase (equivalent to 1 tablet of 600 mg preparation) in postoperative ibuprofen dose was associated with 4.3 MME decrease in postoperative opioid dose requirements. This association supports the use of ibuprofen in postpartum women after cesarean delivery [26]. Both acetaminophen and ibuprofen reduce postoperative opioid consumption and are important components of enhanced recovery pathways [27].

There are several limitations inherent to the current study. The chronic pain conditions were limited only to migraine and chronic back pain. We opted to study these two conditions as back pain and headaches including migraine are the most common chronic pain conditions in the pregnant population [28]. Our study cannot make any inferences about other common chronic pain conditions in the pregnant population, such as pelvic pain or fibromyalgia. Although there have not been major changes in the postcesarean analgesic order set in our institution over the time frame of the current study (2012–2020), variability in prescribing practice in response to the opioid epidemic over the past decade could have affected the observed association. This was a single-center study from an academic medical center in the Pacific Northwest of the United States, and the associations derived would benefit from external validation in other institutions and geographical regions.

In conclusion, our study findings suggest women with chronic back pain and migraine do not report different postpartum pain intensities; however, individuals with preexisting chronic back pain required greater opioid dose than those with migraine during 48 hours after cesarean delivery. Increased postpartum opioid requirement in women with chronic back pain is at a magnitude of difference to be clinically significant and may require postpartum treatment modifications in this patient population. Future studies are required to investigate other chronic pain conditions not evaluated in this study, and the impact of having a combination of chronic pain conditions (chronic back pain plus migraine) which our findings suggest may in combination impact postpartum pain and opioid requirements.

## Figures and Tables

**Table 1:** Demographic, obstetric, intraoperative, and postoperative characteristics.

Characteristic	Migraine (*n* = 465)	Chronic back pain (*n* = 273)	Migraine and chronic back pain (*n* = 43)
Preoperative characteristics			
Age (years)	31.7 ± 5.6	31.4 ± 5.8	32.2 ± 5.6
BMI (kg/m^2^)	33.8 ± 7.8	35.0 ± 9.0	34.8 ± 9.0
Ethnicity (%)			
Caucasian	348 (74.8)	166 (60.8)	27 (62.8)
African American	41 (8.8)	45 (16.5)	7 (16.3)
Asian	28 (6.0)	33 (12.1)	4 (9.3)
Others^a^	48 (10.3)	29 (10.6)	5 (11.6)
Gravidity (%)			
1	179 (38.5)	94 (34.4)	14 (32.6)
2	109 (23.4)	58 (21.3)	11 (25.6)
≧3	177 (38.1)	121 (44.3)	18 (41.9)
Number of previous cesarean deliveries (%)			
0	344 (74.0)	181 (66.3)	23 (53.5)
1	84 (18.1)	54 (19.8)	15 (34.9)
≧2	37 (8.0)	38 (13.9)	5 (11.6)
History of smoking (%)	73 (15.7)	65 (23.8)	11 (25.6)
History of substance use (%)	39 (8.4)	34 (12.5)	9 (20.9)
Marital status (%)			
Married or domestic partner	332 (71.4)	171 (62.6)	25 (58.1)
Divorced or legally separated or single	127 (27.3)	98 (35.9)	16 (37.2)
Unknown	6 (1.3)	4 (0.7)	2 (4.7)
Insurance type (%)			
Private	325 (69.9)	147 (53.9)	27 (62.8)
Public	131 (28.2)	124 (45.4)	15 (34.9)
Unknown	9 (1.9)	2 (0.7)	1 (2.3)
Benzodiazepine use, yes (%)	16 (3.4)	15 (5.5)	1 (2.3)
Antidepressant use, yes (%)	67 (14.4)	41 (15.0)	3 (7.0)
Opioid use, yes (%)	12 (2.6)	24 (8.8)	5 (11.6)
Depression, yes (%)	185 (39.8)	124 (45.4)	14 (32.6)
Anxiety, yes (%)	183 (39.4)	104 (38.1)	14 (32.6)
Intraoperative characteristics			
Labor before delivery (%)			
Labored	234 (50.3)	125 (45.8)	21 (48.8)
Not labored	231 (49.7)	148 (54.2)	22 (51.2)
Emergency status (%)			
Elective	156 (33.6)	107 (39.2)	15 (34.9)
Urgent or emergent	309 (66.5)	166 (60.8)	28 (65.1)
Duration of surgery (minutes)	65 ± 25	69 ± 26	67 ± 21
Intravenous opioids intraoperatively, yes (%)	137 (29.5)	89 (32.6)	13 (30.2)
Intravenous dexamethasone, yes (%)	85 (18.3)	38 (13.9)	9 (20.9)
Postoperative characteristics (0 to 48 hours)			
Acetaminophen dose (mg)	8000 [7000–8000; 0–10000]	8000 [7000–8000; 0–10000]	8000 [7000–8000; 0–8000]
Ibuprofen dose (mg)	4200 [3000–4200; 0–5400]	4200 [2400–4200; 0–5400]	3600 [1800–4200; 0–4800]
Intravenous magnesium, yes (%)	96 (20.7)	44 (16.1)	7 (16.3)

Summary statistics were presented as number (% of patients), mean ± standard deviation, or median (interquartile range; range). Data may not add up to the total number of patients due to missing values. Data may not add up to 100% due to rounding. BMI: body mass index; MME: morphine milligram equivalents; mg: milligram; mcg: microgram. ^a^Others include American Indians or Alaska Natives, Mexican, Mexican American or Chicano, Puerto Rican, Native Hawaiian or other Pacific Islanders, and multiple races.

**Table 2:** Opioid dose requirement and time-weighted average pain scores during 48 hours postoperatively.

	Migraine (*n* = 465)	Chronic back pain (*n* = 273)	Migraine and chronic back pain (*n* = 43)
Postoperative opioid dose in MME	60 [23–120]	90 [37–156]	105 [37–171]
Time-weighted average pain score	3.4 ± 1.4	3.7 ± 1.6	4.1 ± 1.7

Summary statistics were presented as *N* (%) of patients, mean ± SD, or median (IQR). SD: standard deviation; IQR: interquartile range; MME: morphine milligram equivalents.

## Data Availability

The data used to support the findings of this study are available from the corresponding author upon request.
